# Role and regulation of growth plate vascularization during coupling with osteogenesis in tibial dyschondroplasia of chickens

**DOI:** 10.1038/s41598-018-22109-y

**Published:** 2018-02-27

**Authors:** Shu-cheng Huang, Li-hong Zhang, Jia-lu Zhang, Mujeeb Ur Rehman, Xiao-le Tong, Gang Qiu, Xiong Jiang, Mujahid Iqbal, Muhammad Shahzad, Yao-qin Shen, Jia-kui Li

**Affiliations:** 1 College of Veterinary Medicine, Huazhong Agricultural University, Wuhan, 430070 PR China; 2 College of Animal Husbandry and Veterinary Medicine, Tibet Agricultural and Animal Husbandry University, Nyingchi, 860000 Tibet PR China; 3 Bological and Chemical Engineering of Hubei Three Gorges Polytechnic, Yichang, 443000 PR China; 4 University College of Veterinary & Animal Sciences, The Islamia University of Bahawalpur, Bahawalpur, 63100 Pakistan

## Abstract

Tibial dyschondroplasia (TD) is the most-prevalent leg disorder in fast-growing chickens; it is intractable and characterized by abnormal endochondral bone formation of proximal tibial growth-plates (TGPs). Previous studies have shown that bone is a highly vascularized tissue dependent on the coordinated coupling between angiogenesis and osteogenesis, but the underlying mechanisms of bone formation and bone remodeling are poorly defined in TD chickens. Here, we observed that inhibition of vasculogenesis and angiogenesis remarkably impaired vascular invasion in the hypertrophic chondrocyte zone of the TGPs, resulting in the massive death of chondrocytes due to a shortage of blood vessels and nutrients. Moreover, the balance of the OPG (osteoprotegerin)/RANKL (receptor activator of nuclear factor-kB ligand) system is also severely disrupted during the osteogenesis process while coupling with angiogenesis, both of which eventually lead to abnormal endochondral bone formation in TD chickens. Thus, the process of vascular formation in endochondral bone appears to initiate the pathological changes in TD, and improvement of this process during coupling with osteogenesis may be a potential therapeutic approach to treat this intractable disease.

## Introduction

Tibial dyschondroplasia (TD, also known as osteochondrosis), is the most common leg disorder in commercial broilers; it is characterized by non-vascularized and non-calcified cartilage in the proximal tibial growth plates (TGPs), which fails to form endochondral bone and leads to movement reduction and lameness. Surprisingly, approximately 30% of broiler flocks are affected by TD and are likely susceptible to fractures during the feeding process, which can lead to severe economic losses for farmers and can compromise poultry welfare^1–4^. Although these problems are of great concern for researchers worldwide, the underlying etiology of TD remains elusive^5–7^.

Our recent studies performed on chicken models using thiram to induce TD have indicated that the occurrence of this disorder is highly associated with inhibition of bone angiogenesis, resulting in suppression of the TGP development^3^. Consistent with a previous study showing that the occurrence of TD is due to the disruption of genes encoding VEGF receptors followed by subsequent endothelial cell death, which compromises vascularization and the removal of dead chondrocytes and leads to TD lesions^6^. Moreover, Herzog *et al*.^8^ also indicated that the inhibition of proangiogenic factors, such as the activity of heat-shock protein 90 (Hsp90) in the non-vascularized TD-afflicted chicks, resulted in the activation of the angiogenic switch and restored normal growth-plate morphology. Therefore, it can be hypothesized that the inhibition of angiogenesis in TGPs will lead to the occurrence of TD in chickens, whereas adequate vascular invasion in the TGP area can prevent TD lesions^3,6,8^. However, the role of vascular formation in endochondral bone formation and mineralization in TD chickens is still not well defined.

Osteogenesis is critical for the maintenance of a healthy and fully functional skeletal system, which is tightly coupled to the normal growth of blood vessels during endochondral bone formation^9–11^. Blood vessels mediated the transport of circulating cells, oxygen, nutrients and waste products and provide angiogenic and angiocrine signals controlling organ growth and homeostasis^9^. Therefore, normal vascular formation plays a key role in both physiological and pathological processes of the skeletal system. Previous reports have also indicated that the course of new blood vessel formation follows two main processes: vasculogenesis and angiogenesis, which are regulated by a complex network of molecules^12,13^. Among these, vasculogenesis refers to the de novo emergence of a vascular network to initiate the formation of blood islands from mesodermal progenitors to hemangioblasts, followed by migration and association of endothelial cells to form a primitive capillary plexus^14,15^, whereas angiogenesis refers to the generation of vessels by sprouting or non-sprouting from pre-existing capillaries^12^. In this process, a variety of angiogenic factors, including vascular endothelial growth factor (VEGF) and its receptors^16–18^, the fibroblast growth factor (FGF) family^19^, platelet-derived growth factor (PDGF)-BB and its receptor^20,21^, angiopoietin-1 (Ang1), Ang2 and their endothelium-specific receptors such as tyrosine kinase Tie2 (also known as Tek)^22,23^, are all widely expressed as primary inducers of vascular development and postnatal angiogenesis^24,25^. Among these, blockage of the VEGF receptors VEGFR1 and VEGFR2 contributes to decreased blood vessel formation and bone regeneration^17^. However, our understanding of the regulation of these factors in vascular development and formation of the TGP is not well understood.

Additionally, endochondral bone formation is a fundamental mechanism of longitudinal bone growth^26^ and depends on a highly coordinated program of resting chondrocyte differentiation into proliferating chondrocytes, which then hypertrophy and finally undergo apoptotic cell death while being replaced by bone^26,27^. Moreover, bone then undergoes life-long remodeling, which is coordinately regulated by bone-resorbing osteoclasts and bone-forming osteoblasts^28–30^. Numerous studies have indicated that osteoclast formation and activity is regulated by osteoblast lineage-derived receptor activator of nuclear factor-kB ligand (RANKL) and signaling through its receptor, receptor activator of nuclear factor-kappa (RANK), on hematopoietic cells, and this signaling can be inhibited by the decoy receptor osteoprotegerin (OPG), derived from osteoblasts^31–34^. Therefore, this regulatory mechanism of the RANKL/RANK/OPG system is critical for bone remodeling. However, the role of the RANKL/RANK/OPG system in the regulation of bone resorption and bone formation has not been described in TD chickens.

In the present study, we determined whether vasculogenesis and angiogenesis are highly inhibited in TD chickens, which may result in the death of chondrocytes accumulating in the hypertrophic zone of proximal TGPs. Moreover, we found that bone formation is tightly coupled to vascular invasion in proximal TGPs. Thus, our study provides an in-depth mechanism of the pathogenesis of TD, demonstrating osteogenesis inhibition coupled with the suppression of growth plate vascularization.

## Results

### The growth rate of body weight is not coordinated with the growth of bones and blood vessels in chickens

**Figure 1:**
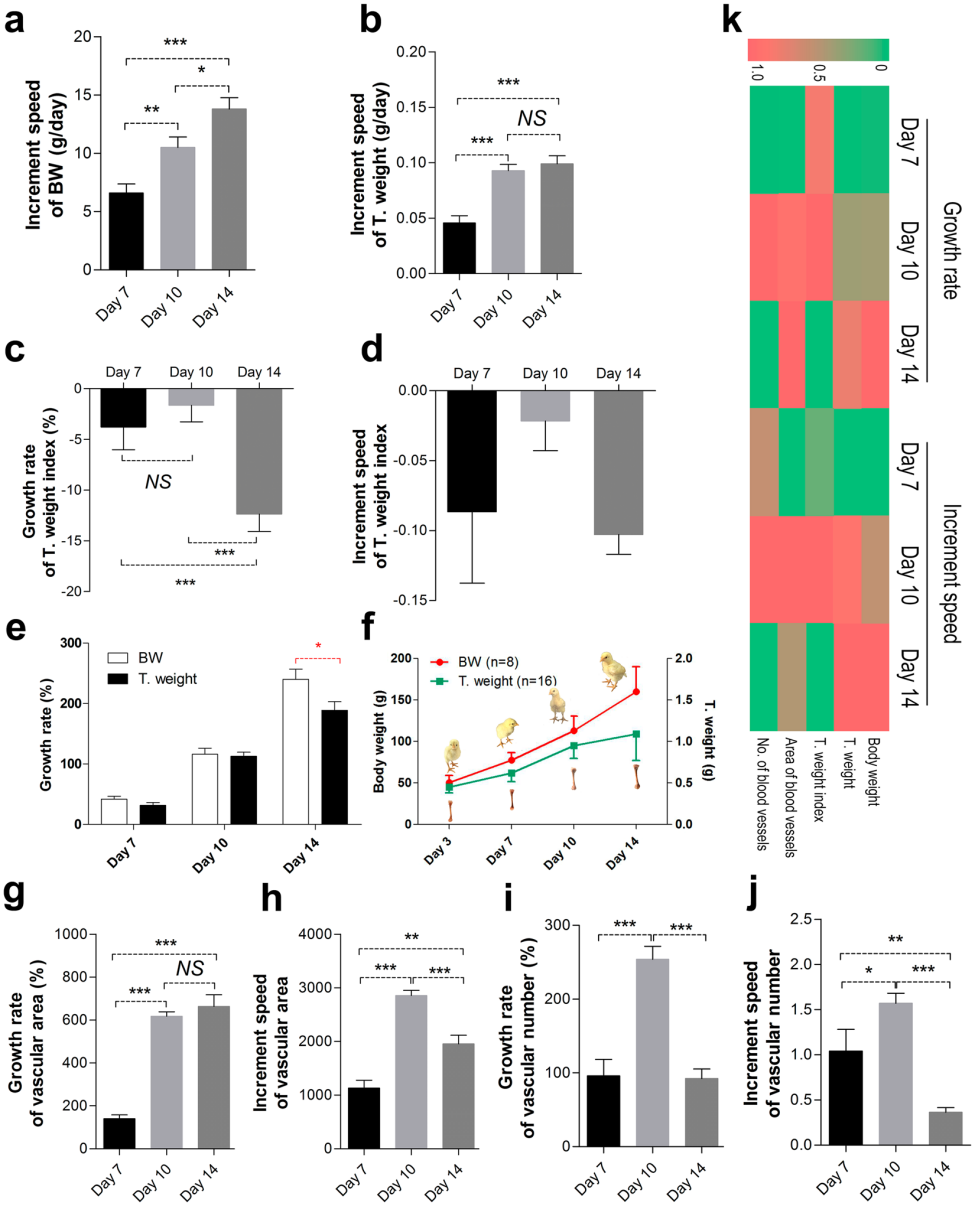
Growth rate of the chicken BW is not in synchronized with the growth of bones and blood vessels. (**a**,**b**) Quantitative analysis of increment speed of BW (**a**; body weight) and T. weight (**b**; tibia weight) in the normal group from 7-, 10 and 14-day-old broiler chickens. (**c**,**d**) Quantitative analysis of growth rate and increment speed of the T. weight index in the normal group from 7-, 10 and 14-day-old broiler chickens. (**e**,**f**) Quantitative analysis of growth rate of BW and T. weight in the normal group from 7-, 10 and 14-day-old broiler chickens. (**g**–**j**) Quantitative analysis of growth rate and increment speed of the vascular area and vascular number in the normal group from 7-, 10 and 14-day-old broiler chickens. N = 8 chickens in each group from four independent experiments; Data represent means ± s.d. ^*^p < 0.05, ^**^p < 0.01, ^***^p < 0.001, one-way analysis of variance (ANOVA) and Least-significant difference (LSD) Duncan test. NS, not significant. (**k**) The changes of BW, T. weight, T. weight index, vascular area and vascular number were shown in a heatmap using the indicated pseudocolor scale from 0 percent (green) to 100 percent (red) relative to average values. Growth rate = (N_t_ − N_0_)/N_0_ * 100%; increment speed = (N_t_ − N_0_)/(t − 0), N: value, t: end time, 0: start time.

The meat-type chicken is a fast-growing breed of poultry, most commonly arbor acre (AA) broilers; its body weight gain can be as high as 2,000 g at 5 weeks old. However, we found that the growth rate and increment of body weight (BW) are more rapid than that of T. weight (Fig. 1a,b,e,f). Moreover, the growth rate and increment speed of the T. weight index was higher on day 10 than day 14 (Fig. 1c,d). This showed that the weight of the legs in AA chickens increases with age. Bone growth and development requires blood vessels to provide nutrients and oxygen. In turn, blood vessels are crucial for normal endochondral bone formation^35^. In addition, we noted that the growth rate of vascular invasion in the hypertrophic chondrocyte zone of proximal TGPs was increased in 14-day-old chickens, albeit without statistical significance, and its increment speed was down-regulated on day 14 (Fig. 1g,h). Moreover, there was a decline in the growth rate and increment speed of the vascular numbers on day 14 (Fig. 1I,j). This suggests that the growth rate of vascular invasion in the hypertrophic chondrocyte zone of proximal TGPs is slower than that of BW, which is in short supply.

Red blood cells (RBCs) and hemoglobin (Hb) are carriers required for the transport of oxygen and nutrients. The change in their number positively correlated with bone mass^36^. Blood parameter measurement showed that the growth rate and increment speed of the RBCs and HGB levels of the broiler chicken decreased with age, which suggested a risk that the oxygen and nutrients in the blood may not be transported efficiently to the chondrocytes and that this defect may become increasingly pronounced with age (Supplementary Fig. 1a-l). Heatmap analysis clearly showed that the growth rate and the increment speed of BW had a marked change (increase); however, T. weight and its index, vascular invasion, were not markedly increased, and even declined (Fig. 1k). Measurement of bone-related parameters is the principal method for the assessment of bone growth^36^. Thus, these findings revealed that broilers are highly susceptible to leg diseases, which may be associated with faster weight gain than bone growth from the legs accompanied with shortage of the vascular invasion^37^.

### Vascular invasion suppression of HZ in chickens with TD

**Figure 2:**
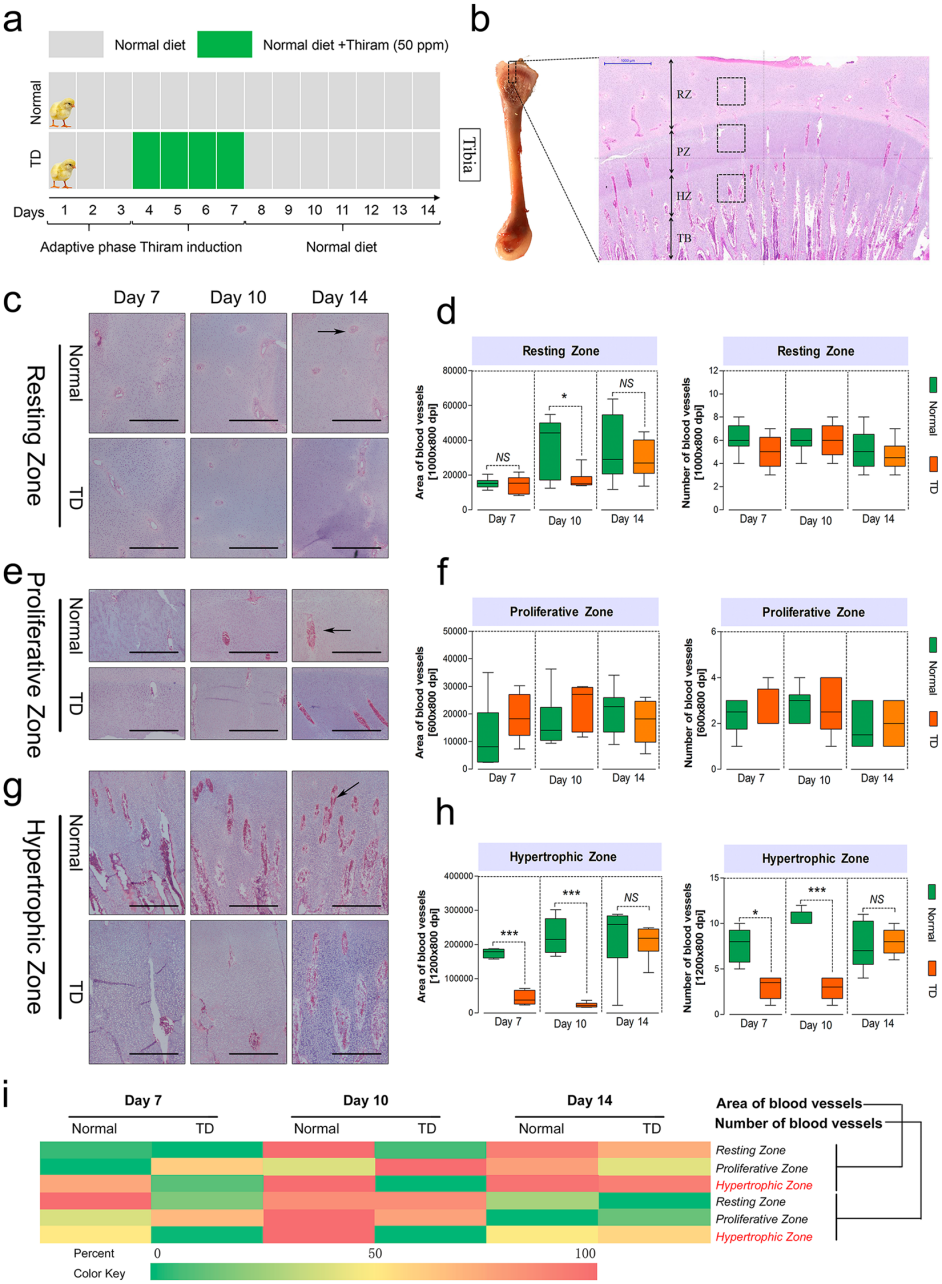
Tibial vascular invasion in the hypertrophic chondrocyte zones is diminished in TD chickens. (**a**) Protocol of thiram treatment in AA chickens (n = 60 chicks/group). (**b**) Schematic diagram of vascular distribution in different zones (RZ, PZ, and HZ) in the proximal tibia. RZ, resting chondrocyte zone; PZ, proliferative chondrocyte zone; HZ, hypertrophic chondrocyte zone; TB, trabecular bone. (**c**,**e**,**g**) Representative images of tibia sections stained with H&E from different zones of the proximal tibia growth plate of normal and TD chickens. Black arrows indicate the blood vessels. Scale bar, 500 μm. (**d**,**f**,**h**) Quantification of tibia blood vessel area and blood vessel number in the RZ, PZ, and PZ, respectively, from normal and TD chickens. Data represent means ± min or max. (n = 6, 3 sample replicates and 2 two isolated statistical groups (3 different microscopic fields were used as an average) using Image-Pro® Plus 6.0), ^*^p < 0.05, ^***^p < 0.001, two-tailed unpaired *t*-test. NS, not significant. (**i**) The changes in area and number of blood vessels were shown in heatmaps using the indicated pseudocolor scale from 0 percent (green) to 100 percent (red), relative to average values.

TD is a common leg problem worldwide with an unknown natural etiology in commercial broilers^2,3,38^. To investigate the underlying mechanisms of the etiology of TD, we initially established a thiram-induced chicken TD model (Fig. 2a). Previous studies have shown that TD has been caused by the accumulation of non-vascularized and non-calcified cartilage masses in the proximal TGPs^2–4^. Histological analysis of tibia was performed first. Interestingly, the vascular invasion showed non-significant changes in the resting chondrocyte zone (RZ) and proliferative chondrocyte zone (PZ) of TD chickens during the experiment. However, both the vascular area and vascular number of the hypertrophic chondrocyte zone (HZ) were remarkably diminished in TD chickens, especially on days 7 and 10. Moreover, heatmap analysis clearly showed that the vascular area and vascular number in the PZ in the TD chicken group (green) was markedly different from the normal group (red), especially on days 7 and 10 (Fig. 2i). These results collectively demonstrate that the occurrence of TD was related to significant suppression of the vascular invasion of the HZ in the proximal TGPs.

### Inhibition of mesodermic differentiation and blood island formation prevent tibial vasculogenesis

**Figure 3:**
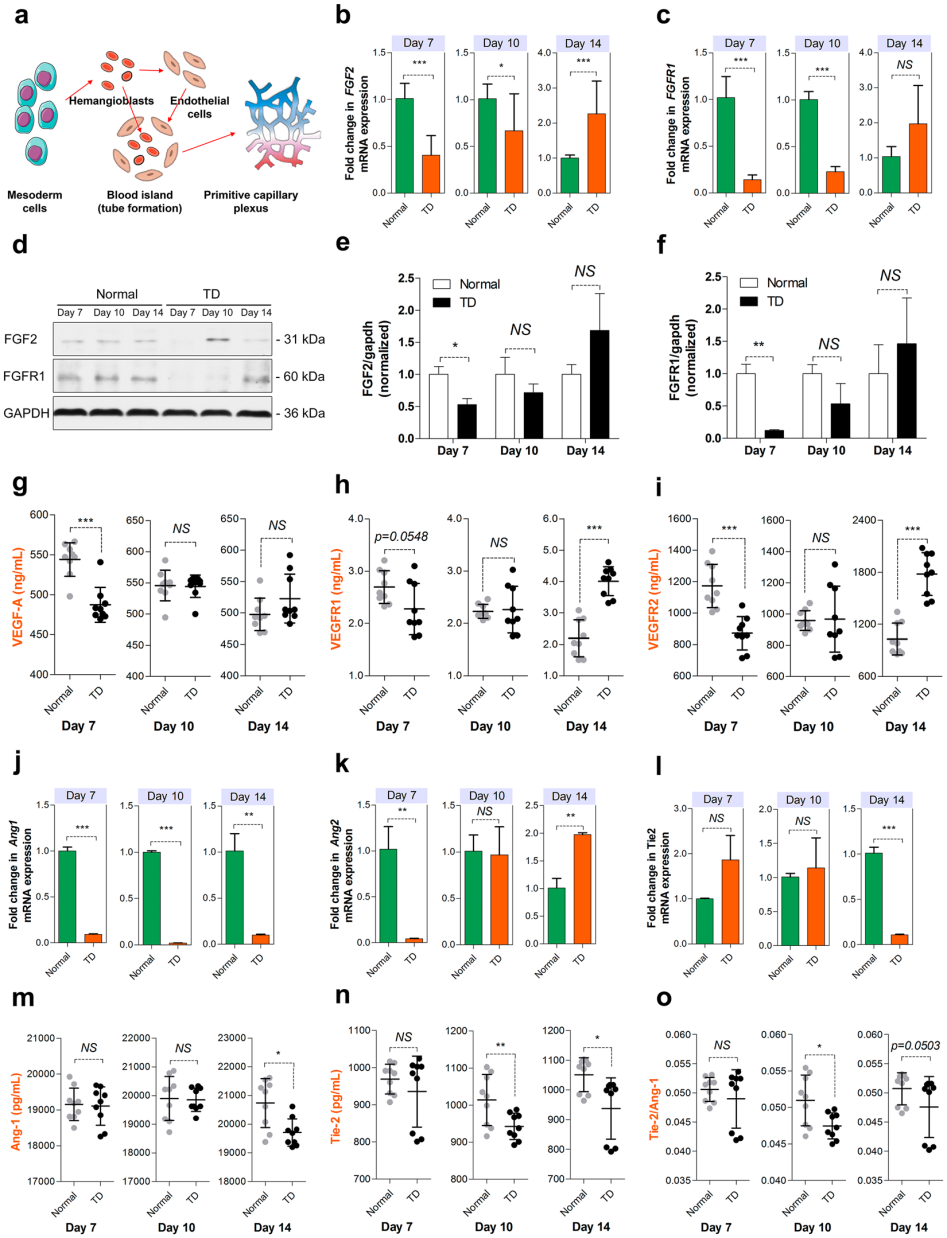
Tibial vasculogenesis is disturbed in TD chickens. (**a**) A schematic diagram illustrating the procedure of vasculogenesis. (**b**,**c**) qRT-PCR analysis of FGF2 and FGFR1 expression levels (normalized to GAPDH) in proximal TGPs sorted from normal and TD chickens at 7, 10 and 14 days, respectively. Data represent means ± s.e.m. (n = 3 biological replicates). ^*^p < 0.05, ^***^p < 0.001, two-tailed unpaired *t*-test. NS, not significant. (**d–f**) Western blot analysis (**d**) and the quantitation (**e**,**f**) of the relative levels of FGF2 and FGFR1 protein expression in proximal TGPs derived from normal and TD chickens at 7, 10 and 14 days, respectively. (n = 3 in each group from three independent experiments). Data represent means ± s.e.m. ^*^p < 0.05, ^**^p < 0.01, two-tailed unpaired *t*-test. NS, not significant. (**g–i**) VEGFA, VEGFR1, and VEGFR2 concentrations assessed by ELISA in serum. (n = 9 in each group from three independent experiments, 3 technical replicates per experiment). Data represent means ± s.d. ^***^p < 0.001, two-tailed unpaired *t*-test. NS, not significant. (**j–l**) qRT-PCR analysis of Ang1, Ang2 and Tie2 expression (normalized to GAPDH) levels in proximal TGPs sorted from normal and TD chickens at 7, 10 and 14 days, respectively. Data represent means ± s.e.m. (n = 3 biological replicates). ^**^p < 0.01, ^***^p < 0.001, two-tailed unpaired *t*-test. NS, not significant. (**m**–**o**) Ang1 and Tie2 concentrations assessed by serum ELISAs and Tie2/Ang1 ratios were determined. (n = 9 in each group from three independent experiments, 3 technical replicates per experiment). Data represent means ± s.d. ^*^p < 0.05, ^**^p < 0.01, two-tailed unpaired *t*-test. NS, not significant.

Based on the above findings, understanding the mechanism of the vascular invasion suppression in the HZ is important for understanding the pathogenesis of the chicken TD. Therefore, we tried to investigate the molecular mechanism of blood vessel development in the proximal tibial hypertrophy zone. Clearly, new blood vessel formation proceeds by two main processes, vasculogenesis and angiogenesis, which are regulated by a complex network of molecules^12^. Vasculogenesis refers to the determination and differentiation of endothelial progenitor cells from mesodermal blood islands consisting of hemangioblasts and endothelial cells, and their de novo organization by the interaction with the recruitment of peripheral cells into a primitive capillary plexus (Fig. 3a)^12–15^.

Thereafter, to investigate the role of related genes in regulating the vasculogenesis process, we collected tibia specimens and sera from normal and TD chickens, and then examined the changes in the mRNA and protein expression levels at 7, 10 and 14 days of age, respectively. Compared with those in the normal group, qRT-PCR analysis showed that the levels of the mesodermic differentiation marker genes (FGF2 and FGFR1) in proximal TGPs were significantly lower in TD chickens at 7 days of age^19,39,40^, and then markedly recovered at 10 and 14 days, when thiram treatment was removed (Fig. 3b,c). Moreover, Western blot analysis further showed that the protein levels of FGF2 and FGFRR1 had similar changes in TD chickens compared to those in normal chickens (Fig. 3d–f). Importantly, we found that the protein level of the blood island formation marker gene VEGFA and its receptors remarkably decreased in the serum of TD chickens^16^. Moreover, inhibition of protein levels of VEGFA and its receptors decreased when thiram was removed from the TD chicken group at 10 and 14 days of age (Fig. 3j–i). On the other hand, we noticed that there was a more marked down-regulation of VEGFR2 than VEGFR1 (Supplementary Fig. 2a,b).

Blood islands further mature into primitive capillary plexi, which requires the regulation of related genes (including Ang1, Ang2 and their endothelium-specific receptor tyrosine kinase Tie2; moreover, Ang-2 is a naturally antagonist for Ang1 and its Tie2)^22,23^. We next determined whether the maturation of tube structures with peripheral cell interaction from blood islands was also inhibited in TD chickens; we then examined the Ang1, Ang2 and Tie2 mRNA levels in proximal TGPs by qRT-PCR. Consistently, we also found that the levels of Ang1 and Ang2 in proximal TGPs were significantly lower in the TD chickens at 7 days old, which markedly recovered at 10 and 14 days as thiram-treatment was removed, compared with those in the normal group (Fig. 3j,k). In contrast, the levels of Tie2 were dramatically higher at 7 and 10 days, and then remarkably reduced at 14 days in TD chickens compared to those in normal chickens (Fig. 3l). In addition, ELISA results further showed that the protein levels of Ang1 and Tie2 in the serum were obviously inhibited in TD chickens (Fig. 3m–o). Based on these results, we propose that inhibition of mesodermic differentiation, blood island formation and primitive capillary plexus maturation-related gene expression contributes to the delay of vasculogenesis process, followed by the occurrence of TD or other leg diseases in chickens.

### Inhibition of vascular proliferation inhibits tibial angiogenesis

**Figure 4:**
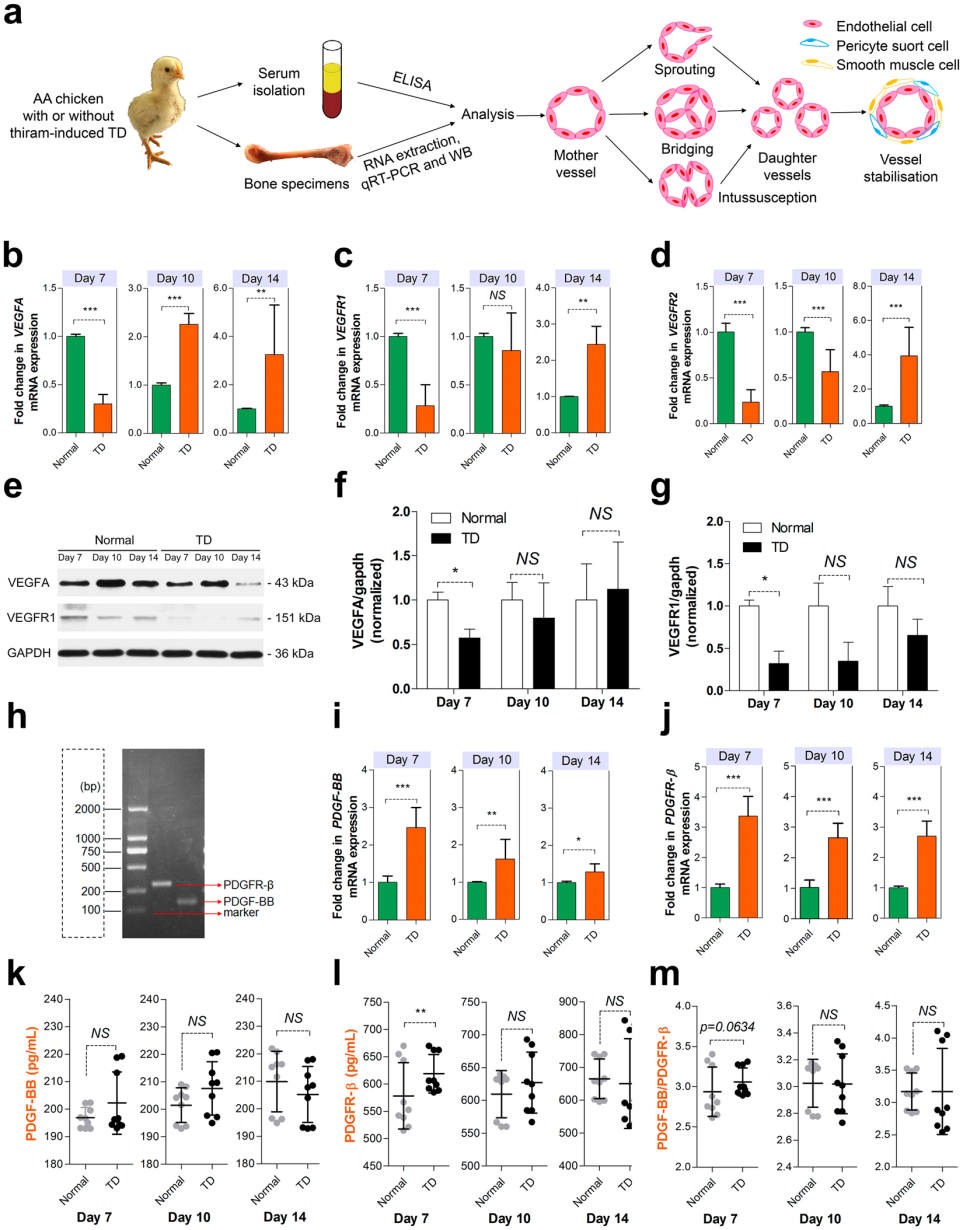
Inhibition of tibial angiogenesis in TD chickens. (**a**) A schematic diagram illustrating the experimental design and procedure of angiogenesis. Serum and bone specimens were collected from normal and TD chickens at 7, 10 and 14 days old, respectively. (**b–d**) qRT-PCR analysis of VEGFA, VEGFR1 and VEGFR2 expression (normalized to GAPDH) in proximal TGPs sorted from normal and TD chickens at 7, 10 and 14 days, respectively. Data represent means ± s.e.m. (n = 3 biological replicates). ^**^p < 0.01, ^***^p < 0.001, two-tailed unpaired *t*-test. NS, not significant. (**e–g**) Western blot analysis (**e**) and the quantitation (**f**,**g**) of the relative levels of VEGFA and VEGFR1 protein expression in proximal TGPs derived from normal and TD chickens at 7, 10 and 14 days, respectively. (n = 3 in each group from three independent experiments). Data represent means ± s.e.m. ^*^p < 0.05, two-tailed unpaired *t*-test. NS, not significant. (**h**) The gel electrophoresis diagram of the PDGF-BB and PDGFR-β gene. (**i**,**j**) qRT-PCR analysis of PDGF-BB and PDGFR-β expression levels (normalized to GAPDH) in proximal TGPs sorted from normal and TD chickens at 7, 10 and 14 days, respectively. Data represent means ± s.e.m. (n = 3 biological replicates). ^***^p < 0.001, two-tailed unpaired *t*-test. NS, not significant. (**k–m**) PDGF-BB and PDGFR-β concentrations assessed by serum ELISAs and PDGF-BB/PDGFR-β ratios were also determined. (n = 9 in each group from three independent experiments, 3 technical replicates per experiment). Data represent means ± s.d. ^**^p < 0.01, two-tailed unpaired *t*-test. NS, not significant.

Angiogenesis is crucial for the proliferation and maturation of blood vessels. To further ascertain whether angiogenesis has similar changes in TD chickens compared to normal chickens, we collected tibia specimens and sera from normal and TD chicken groups, then examined the changes in mRNA and protein expression levels at 7, 10 and 14 days of age, respectively, to help us understand the process of angiogenesis from the mother vessel to the daughter vessels by sprouting or non-sprouting from pre-existing capillaries (Fig. 4a). VEGF and its receptors (including VEGFR1 and VEGFR2) not only have an effect on the vascular differentiation but also play a key role in vascular proliferation^16–18^. qRT-PCR further confirmed that the levels of the VEGFA and its receptors in proximal TGPs were significantly lower in TD chickens at 7 days old, which markedly recovered at 10 and 14 days of age due to the removal of thiram compared with the levels in the normal group (Fig. 4b–d). Moreover, Western blot analysis further revealed that the levels of VEGFA and VEGFR1 proteins had a similar change in TD chickens compared with normal chickens (Fig. 4e–g).

PDGF-BB and activation of its receptor PDGFR-β are essential for vessel stabilization and are also responsible for pericyte support cell recruitment and interaction with smooth muscle cells^20,21^. To gain further insights into the molecular mechanisms by which PDGF-BB and its receptor PDGFR-β regulate vascular stabilization and maturation, we analyzed the expression levels and protein concentrations of PDGF-BB and PDGFR-β in the proximal TGPs and sera of TD chickens. Interestingly, PDGF-BB and PDGFR-β genes, 158 bp and 308 bp, respectively, were used, and the levels of the PDGF-BB and PDGFR-β in proximal TGPs were remarkably higher in the TD chicken group at 7 days of age than those in the normal chicken group (Fig. 4h–j). Furthermore, the serum PDGF-BB and PDGFR-β protein levels in TD chickens were also higher than those in normal chickens (Fig. 4k–m). The above data suggested that vascular proliferation inhibition and vascular maturation promotion could provide a greater impact on vascular distribution in the hypertrophic chondrocyte zones of proximal TGPs via inhibition of VEGFA and its receptors expression and promoting PDGF-BB and PDGFR-β up-regulation.

### Growth plate vascularization is essential for chondrocyte proliferation and differentiation to promote bone mineralization

**Figure 5:**
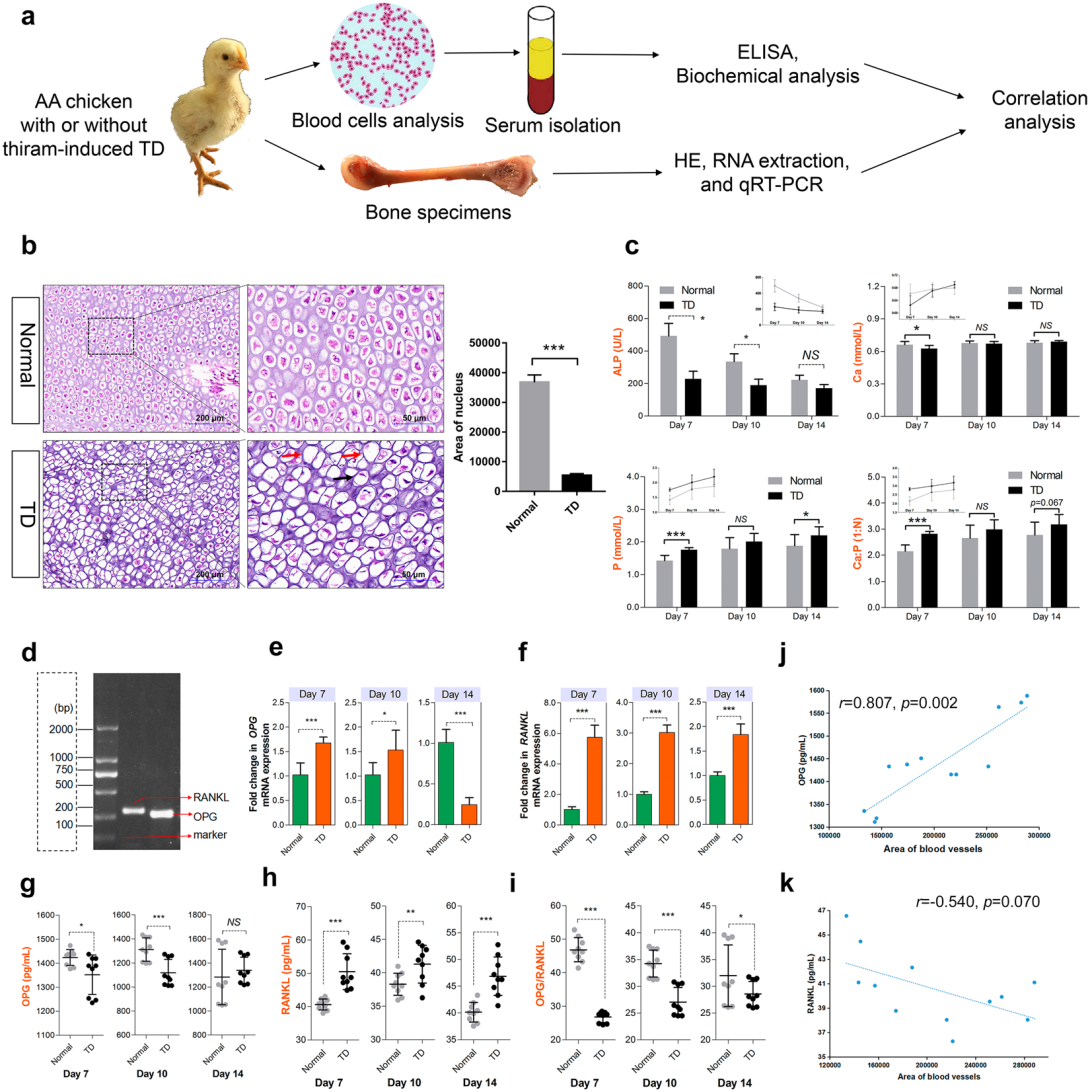
Growth plate vascularization is essential for chondrocyte growth and differentiation to promote bone mineralization. (**a**) A schematic diagram illustrating the experimental design. Serum and bone specimens were collected from normal and TD chickens at 7, 10 and 14 days old, respectively. (**b**) Representative images of tibia sections stained with H&E from lower hypertrophic chondrocyte zone of the proximal tibia growth plate of normal and TD chickens. Higher magnification is shown on the right. Black arrow indicates pyknosis. Red arrows indicate karyolysis. (**c**) Serum alkaline phosphatase (ALP), calcium (Ca) and phosphorus (P) levels in normal and TD chickens. Data represent means ± s.e.m. (n = 3 chickens from three independent experiments). ^*^p < 0.05, ^***^p < 0.001, two-tailed unpaired *t*-test. NS, not significant. (**d**) Gel electrophoresis diagram of the OPG and RANKL genes. (**e**,**f**) qRT-PCR analysis of OPG and RANKL expression (normalized to GAPDH) in proximal TGPs sorted from normal and TD chickens at 7, 10 and 14 days, respectively. Data represent means ± s.e.m. (n = 3 biological replicates). ^**^p < 0.01, ^***^p < 0.001, two-tailed unpaired *t*-test. NS, not significant. (**g–i**) OPG and RANKL concentrations assessed by serum ELISAs and the OPG/RANKL ratio were also determined. (n = 9 in each group from three independent experiments, 3 technical replicates per experiment). Data represent means ± s.d. ^**^p < 0.01, two-tailed unpaired *t*-test. NS, not significant. (**j**,**k**) Correlation analysis between serum OPG level (j, n = 9), serum RANKL level (k, n = 9) and area of blood vessels in the hypertrophic chondrocyte zone of proximal tibia, respectively, were determined by Spearman tests. (n = 6, 3 sample replicates and 2 two isolated statistical groups (3 different microscopic fields were used as an average)).

To gain insights into how angiogenesis inhibition in the hypertrophic chondrocyte zone of proximal TGPs delays bone mineralization, we compared the morphology of chondrocytes of TD chickens with that in normal chickens. Pathological analysis of the TGPs from 7-day-old chickens showed pyknosis and karyolysis of lower hypertrophic chondrocytes and a significantly reduced nucleus area of hypertrophic chondrocytes in TD chickens, which was most evident in hypertrophic chondrocyte necrosis (Fig. 5b), resulting in a significantly thicker growth plate due to a reduction of blood vessels, which may prevent or reduce the removal of necrotic chondrocytes from the hypertrophic chondrocyte zone compared to normal chickens (Fig. 2g,h)^6^. Moreover, a recent study has shown that the total RBC counts, Hb concentration and Hct values at 7 days in the TD chicken group are clearly lower than those in normal chickens^3^. Even MCH (mean corpuscular hemoglobin) and MCHC (mean corpuscular hemoglobin concentration) were lower, whereas no significant differences in MCV (mean corpuscular volume) were found between TD chickens and normal chickens by complete blood counts (CBCs), as shown in Supplementary Fig. 3a–c. These results suggest that the death of chondrocytes is not only associated with the decline of vascular invasion in the hypertrophic chondrocyte zones but also with the reduction of RBCs and Hb, which transport oxygen and nutrients.

To further determine whether the thickened growth plate was directly associated with dead chondrocytes that failed to complete calcification and accumulated in hypertrophic chondrocyte zones due to reduced vascularization, we consistently collected tibia specimens, whole blood and sera from normal and TD chickens (Fig. 5a). Serum biochemical parameters showed that alkaline phosphatase (ALP) activity was markedly inhibited, calcium (Ca) levels were also markedly decreased, and phosphorus (P) levels were prominently increased in 7-day-old TD chickens compared with normal chickens. In addition, we also found that the serum Ca/P ratio was significantly changed (decreased) in 7-day-old TD chickens compared with those in normal chickens (Fig. 5c). These results imply that the normal formation of the bone may be disturbed due to the disruption of the Ca and P balance in the serum.

RANKL signaling can regulate osteoclast formation, activation and survival in normal bone modeling and remodeling, even in a variety of pathologic conditions characterized by increased bone turnover^34^. In contrast, OPG can protect bone from excessive resorption by binding to RANKL and preventing it from binding to RANK^31^. Thus, the RANKL and OPG levels in bone turnover are major determinants of the bone mass and strength^31,33,34,41^. To investigate whether the molecular mechanism of bone formation is inhibited in TD chickens through the destruction of the OPG/RANKL system, we examined OPG and RANKL (266 bp and 298 bp, respectively) mRNA and protein levels by qRT-PCR and ELISA (Fig. 5d). The results showed that the levels of the OPG in proximal TGPs were elevated at 7 days, and then significantly decreased at 14 days in TD chickens compared with those in the normal group (Fig. 5e), whereas RANKL was overexpressed in TD chickens compared those in normal chickens (Fig. 5f). Likewise, the changes in OPG and RANKL protein levels in whole serum were almost similar to their mRNA expression in proximal TGPs of TD chickens (Fig. 5g,h). In addition, we further compared the levels of OPG and RANKL in serum and found that the balance between the OPG/RANKL parameters was disrupted (Fig. 5i). Furthermore, the serum OPG level was positively correlated with vascular area (Fig. 5j). However, we found a negative correlation between serum RANKL level and vascular area in the hypertrophic chondrocyte zone of proximal TGPs (Fig. 5k). Taken together, these data indicate that the formation of bone is inhibited via disruption of the balance of the Ca/P ratio and OPG/RANKL system, which are associated with reduced vascular distribution in the hypertrophic chondrocyte zones of proximal tibias.

### Suppression of tibia structure and tibia mass are linked to osteogenesis

**Figure 6:**
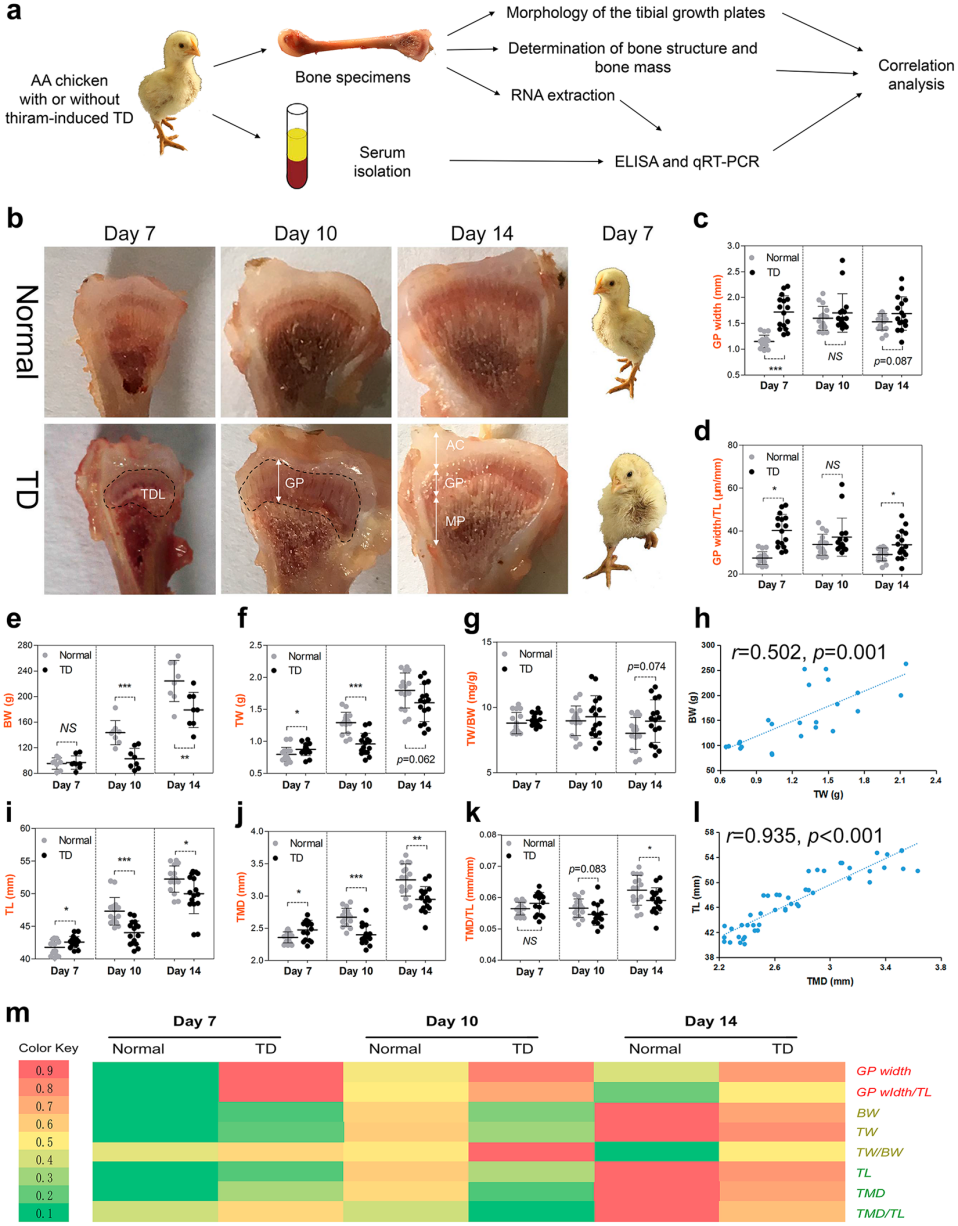
Suppression of tibia structure and tibia mass are linked to osteogenesis. (**a**) A schematic diagram illustrating the experimental design. Sera and bone specimens were collected from normal and TD chickens at 7, 10 and 14 days old, respectively. (**b**) Representative morphological images of proximal tibias from normal and TD chickens. Real images on the right represent normal broiler chicken (above) and lameness of the broiler chicken (below). TDL, Tibial dyschondroplasia lesion; AC, articular cartilage; GP, growth plate; MP, metaphysis. (**c**,**d**) Quantitative morphological analysis of tibia mass in normal and TD groups from 7-, 10 and 14-day-old broiler chickens. (GP width, growth plate width; GP width/TL, growth plate width per tibia length. N = 8 chickens in each group from four independent experiments). Data represent means ± s.d. ^*^p < 0.05, ^***^p < 0.001, two-tailed unpaired *t*-test. NS, not significant. (**e–g**, **i–k**) Quantitative analysis of tibia structure and tibia mass in normal and TD groups from 7-, 10, and 14-day-old broiler chickens. (BW, body weight; TW, tibia weight; TW/BW, tibia weight per body weight; TL, tibia length; TMD, tibia mid-diameter; TMD/TL, tibia mid-diameter per tibia length. N = 8 chickens in each group from four independent experiments). Data represent means ± s.d. ^*^p < 0.05, ^**^p < 0.01, ^***^p < 0.001, two-tailed unpaired *t*-test. NS, not significant. (**h**,**l**) Correlation analysis between BW and TW (**h**, n = 8), between TL and TMD (**l**, n = 8) in normal and TD chickens were determined by Spearman tests. (**m**) The changes in tibial growth plate parameters and tibia parameters were shown in a heatmap using the indicated pseudocolor scale from 0.1 percent (green) to 0.9 percent (red) relative to average values.

To determine whether inhibition of bone calcification could inhibit normal bone formation, we consistently collected tibia specimens and whole sera from normal and TD chickens (Fig. 6a). First, we undertook a morphological analysis of the TGP and measured the width of the growth plate. The results showed that the width of the growth plate (GP) and GP width/ TL (tibia length) ratio were significantly increased. Furthermore, we also observed that the area of TD lesions was significantly increased at 7 days in TD chickens (Fig. 6b–c). This further confirms that the growth plate width is increased, which may be related to the dead chondrocytes clustered in the growth plate area due to the lack of blood vessels (Fig. 5b).

Quantitatively, bone histomorphometry analysis showed that the TW (tibia weight), TL (tibia length) and TMD (tibia mid-diameter) were all slightly higher at 7 days due to swollen joint, then significantly reduced at 10 and 14 days in TD chickens compared to normal chickens (Fig. 6f,I,j). In addition, BW showed a similar change in the TD chickens (Fig. 6e). In contrast, TW/BW (tibia weight to body weight ratio) and TMD/TL (tibia mid-diameter to tibia length ratio) were slightly increased during the experiment (Fig. 6g,k). Moreover, we found a positive correlation between BW and TW (Fig. 6h) and between TMD and TL (Fig. 6l). A heatmap clearly showed that the GP width and the GP width/TL ratio had a significant increase in the 7-day-old TD chickens (red) compared to the normal chickens (green). In addition, the heatmap also clearly showed a similar change in the BW, TW, TL and TMD (Fig. 6m). We presented bone parameters-related evidence to demonstrate that the bone mass-related parameters (BW, TW and TW/BW) and the bone structure-related parameters (TL, TMD and TMD/TL) were inhibited in TD chickens. Interestingly, the serum OPG level was positively correlated with the TW and TL, respectively (Supplementary Fig. 4a,b), whereas we found a negative correlation between serum RANKL level and TW and TL, respectively. (Supplementary Fig. 4c,d). Collectively, these results indicate that the suppression of the tibia structure and tibia mass eventually leads to the bone formation inhibition in TD chickens.

## Discussion

Using a chicken model of thiram-induced TD, we performed a series of *in vivo* studies found that the regulation of the vascular formation is strongly inhibited in TD chickens, leading to the impairment of bone vascularization in the hypertrophic zone of TGPs. In addition, endochondral bone formation is also inhibited by disrupting the balance of the Ca/P ratio and the OPG/RANKL system to suppress tibia structure and tibia mass. Mechanistically, the inhibition of vasculogenesis and angiogenesis showed significant impairment in the vascular invasion of the hypertrophic zone of the TGP, resulting in the massive death of chondrocytes due to a shortage of blood vessels and nutrients. In addition, the balances of the Ca/P ratio and OPG/RANKL system are also disrupted, leading to osteogenesis disorders during coupling with angiogenesis, both of which ultimately may lead to abnormal bone formation (TD) in chickens.

TD, ascites syndrome and sudden death syndrome are the three major nutritional metabolic diseases that seriously affect the modern poultry breeding industry. Fast-growing broiler chickens are particularly susceptible to TD^42,43^. However, the cause of the disease has remained unknown. The comparative results of the BW and tibia parameters showed that the overall weight gain was faster than the tibial growth and vascular distribution increase in the hypertrophic chondrocyte zone of the TGP. Moreover, production speed of the RBC and Hb decreased with age, which indicates that the oxygen and nutrients in the blood may not be efficiently transported to the chondrocytes. Therefore, the growth rate of BW is not synchronized with that of the bones and blood vessels, which is the main trigger for TD^37^. Our previous studies have found that no case of TD has been reported in Tibetan chickens, which have abundant vascular distribution in the TGP^44^. Increasing evidence suggests that compression vascularization could result in the occurrence of TD, whereas activation of the angiogenic switch could reinstate normal growth-plate morphology, contributing to TD recovery^6,8^. Thus, blood vessel invasion from the metaphyseal region is essential for the formation of endochondral bone^9,45,46^.

Limited reports are available concerning the role of blood vessels in endochondral bone formation in chickens^6,8,47^. Our previous studies have found that angiogenesis-related gene inhibition from TGPs could lead to the suppression of vascular formation in the hypertrophic chondrocyte zone, which is closely associated with the occurrence of TD^3^. However, the underlying molecular mechanisms of vascular formation in TD chickens has not yet been comprehensively investigated. Further study has found that TD chickens have delayed vasculogenesis processes via inhibition of mesodermic differentiation, blood island formation and primitive capillary plexus maturation-related gene expression, which subsequently inhibits angiogenesis through impairment of vascular invasion in the hypertrophic chondrocyte zone of proximal TGPs by inhibiting vascular proliferation and promoting vascular maturation (Figs 3 and 4). As bone vasculature is required for osteogenesis and plays a vital role in bone development and remodeling^46^, the reduction of tibial vascularization may be a direct cause of TD.

However, whether the decrease of tibial vascularization affects endochondral bone formation has not been evaluated. We noted that TD lesions mainly occur in the hypertrophic zone, showing a pale, non-vascular region (Fig. 2g). A shortage of blood vessels may be a consequence of dead chondrocytes in the hypertrophic zone due to a decrease in the supply of oxygen and nutrients^45^. This theory was supported by our findings that thiram-induced TD chicken showed pyknosis and karyolysis of hypertrophic chondrocytes and down-regulation of VEGFA and its receptors (Fig. 4b–g; Fig. 5b), which was most evident in hypertrophic chondrocyte necrosis^48^. Moreover, hypertrophic chondrocytes are also a main contributor to skeletal growth, and can release several cytokines such, as VEGF, to stimulate angiogenesis through the activation of VEGF receptors in endothelial cells^27,49,50^. In addition, the decrease in vascular invasion also affects the recruitment of osteoclasts and osteoblasts, as well as the apoptosis of terminal hypertrophic chondrocytes, resulting in the failure of replacement of mineralized cartilage with bone^9,46,49,51^, which may be because osteoclasts are responsible for the breakdown or resorption of bone^52^, and osteoblasts are responsible for the synthesis and mineralization of bone as well as modulation of the differentiation of osteoclasts^53^. Therefore, decreased tibial vascularization not only causes the death of hypertrophic chondrocytes but also inhibits bone mineralization by interfering with bone resorption and bone formation.

The OPG/RANKL/RANK cytokine system is essential for osteoclast biology^31,33,34^. Various studies have suggested that human metabolic bone diseases are associated with alterations of this system^33,54–56^. The balance between the expression levels of the RANKL (stimulator of osteoclastogenesis) and OPG (inhibitor of osteoclastogenesis) dictates the quantity of bone resorbed^34,57^. Alternatively, imbalance of the RANKL/OPG ratio could lead to an uncontrolled loss of bone mass^56,58^. For the first time, we have demonstrated that the resorption and formation of bone is changed due to the disruption of the balance of the Ca/P ratio and the OPG/RANKL system, resulting in suppression of the tibia structure and tibia mass in TD chickens. Moreover, we also found that the OPG protein level, Ca concentration and ALP activity in serum were all remarkably diminished, which further verifies that bone turnover is inhibited in TD chickens^59^. Similarly, a previous study by Simonet *et al*.^60^ indicated that animals lacking OPG have accelerated osteoclastogenesis and develop severe osteoporosis. Taken together, these results suggest that osteogenesis inhibition is closely coupled with angiogenesis impairment, which contributes to the inhibition of tibial bone formation and leads to the occurrence of TD through alterations of the Ca/P ratio and OPG/RANKL system.

In summary, our study found that adequate blood vessels are extremely important and essential for normal bone formation. In TD chickens, the formation of blood vessels is not only disturbed, but the formation of bone is also inhibited. A decrease in vascular invasion may lead to the death of chondrocytes and their accumulation in the growth plate hypertrophic zone, which contributes to the failure of proliferation and differentiation of chondrocytes and the subsequent failure of bone mineralization.

## Materials and Methods

### Chicken husbandry

All AA chickens (one-day-old; chicks were used regardless of gender) were purchased from a commercial hatchery (Chia Tai Animal Husbandry Co. Ltd., Wuhan, China). The chicks were reared in two-layer metal cages for 14 days using a recommended standard brooding temperature (from 33 °C~35 °C during the first week, steadily reduced to 29 °C at the end of the second week) and hygienic conditions. Daily lighting was fixed at a 23 h/1 h light/dark cycle throughout the entire experimental period. Additionally, feed and water were provided *ad libitum*.

Animal experiments were approved by the Ethical Committee of the Huazhong Agricultural University (Permit No. 4200695757) and performed based on the state guidelines from the Laboratory Animal Research Centre of Hubei province in China.

### TD establishment

AA chickens were randomly divided into two separate groups: the normal chicken group (normal diet) and the thiram-induced TD chicken group (a normal diet and a normal diet plus 50 mg/kg of thiram, respectively; n = 60/group, with 15 chicks per replicate and 4 replicates per group). The experiment was performed over 14 days, and thiram (tetramethyl thiuram disulfide, #C10036460, Macklin Biochemical Co., Ltd. Shanghai, China) was added on day 3 and removed on day 7, followed by feeding with a normal diet until the end of the experiment (Fig. 2a).

### Tibial growth plate collection

Eight chicks from the normal- and TD-chicken groups, respectively, at days 3, 7, 10 and 14 of the experiment were randomly chosen for examination. All group chickens were euthanized by cervical dislocation before injection of pentobarbital (25 mg/kg). The tibia specimens were retrieved, and growth plates were detached from articular cartilage of the proximal tibia via dissection with a surgical knife and were instantly frozen in liquid nitrogen and stored at −80 °C for further qRT-PCR and Western blot analysis, as previously described^3,61^.

### Measurement of tibial parameters

Each chick per group selected for the next experiment was weighed, and the value was recorded as the body weight (BW). Next, all chicks were sacrificed, and tibial specimens (n = 16) were collected for analysis of tibia weight (TW, mg), tibia Length (TL, mm), tibia mid-diameter (TMD, mm) and growth plate width (GP width, mm). These bone histomorphometric parameters were determined by an electronic balance sensitive to 0.001 g and Digital Calipers (#SATA91511, TATA Company, Shanghai, China), respectively. In addition, tibia weight index (T. weight index, determined as the TW per BW for each bird, mg/g), GP width/ TL ratio and TMD/TL ratios were calculated.

### Blood parameter assays

Before euthanasia, blood samples were obtained through the wing veins using heparinized syringes. Red blood cell (RBC) counts, hemoglobin (Hb) levels, hematocrit (Hct) values, mean corpuscular volume (MCV), mean corpuscular hemoglobin (MCH) and mean corpuscular hemoglobin concentration (MCHC) from all groups were determined using an automatic blood analyzer (#XFA6000, Pulang Company, Nanjing, China).

### Biochemical parameter assays

The blood samples were centrifuged at 3,000 × g for 20 min for the separation of serum and stored at −20 °C until subsequent use and further analysis to determine the ALP, Ca and P serum levels. ALP (#160621), P (#170711) and Ca (#170711) reagent kits were purchased from BioSino Bio-Technology and Science Inc. (Beijing, China) and were performed to measure the serum levels of ALP, Ca and P in both the normal- and TD-chicken group via a semi-automatic biochemical analysis system (#GF-D800, Gaomi Caihong Analytical Instruments Co., LTD, Shandong, China), according to the manufacturer’s instructions.

### Histology of the tibial growth plates

As previously described, the stripping of the tibial longitudinal muscles and preparation of sagittal sections of the proximal TGPs were performed to analyze the morphology^60^. Collected tibial specimens (n = 3) were fixed in 4% paraformaldehyde at 4 °C in PBS and decalcified in 10% EDTA. After the specimens were dehydrated in ethanol and cleared in xylene, all tibial specimens were embedded in paraffin, and histological sections 4~5 μm thick were prepared and stained with hematoxylin and eosin (H&E) for microscopic examination as previously described^2,42^. In addition, the areas of blood vessels and numbers of blood vessels in the hypertrophic zone adjacent to the TGP (3 chicks/group, three different microscopic fields/chick) were counted with two isolated groups using professional image analysis software (#Version 6.0.0.260, Image-Pro® Plus, Media Cybernetics, Inc.)^3,62^.

### RNA extraction and quantitative real-time PCR (qRT-PCR) analysis

Total tibial growth plate RNA was extracted using the TRIzol reagent (#119701, Life Technologies, Carlsbad, California, USA). cDNA was synthesized using a cDNA Synthesis Kit (#K10624, TransBionovo Co., Ltd, Beijing, China) per the instructions of the manufacturer. qRT-PCR was performed with the *TransStart* Tip Green qPCR Kit (#L10227, TransBionovo Co., Ltd, Beijing, China) in a Step One-Plus^TM^ Real-Time PCR System (#271002707, Applied Biosystems, CA, USA). All reactions were performed in triplicate. GAPDH was used as an internal control, and the relative expression of each target gene was normalized to the expression of GAPDH by the ∆Ct method. Primer sequences of the qRT-PCR primers are listed in Table 1.

**Table 1:** Primers used for qRT-PCR analysisF.

Gene	Sequence (5′-3′)
GAPDH	F: CCTCTCTGGCAAAGTCCAAG R: GGTCACGCTCCTGGAAGATA
FGF2	F: AAACCGCTTTCTGGCTATGAA R: AGTGCCACATACCAATCAGA G
FGFR1	F: GGAGCGAGACCACCTACTTC R: GGCATAGCGGACCTTGTAC
VEGFA	F: CGATGAGGGCCTAGAATGTGTC R: AGCTCATGTGCGCTATGTGC
VEGFR1	F: TGTAACTAAGTATGCCTGTGG R: GGAGTTGTTGGGTATCTGC
VEGFR2	F: GCCAACTCTATGGCAGAAGC R: CTGAACACCATGCCACTGTC
Ang1	F: ACTGTCCATCCTCCTCCA R: GCGGCGTCTACACTATTT
Ang2	F: GTGGCAGCGTGGATTTTCA R: GCGAATCATCATAGTCGTGGCTT
Tie2	F: CAGTCACAGCAAACAAG R: AAGCAGAAGTAAAGAGG
PDGF-BB	F: CCCATACCCGAAGATA R: CGAGACAGGGACACAT
PDGFR-β	F: AAGTGGCAGTCAAGAT R: CAGACAAGGAGAGGTG
OPG	F: AGACTGGAACAGCAACGACGAG R: GACAGACTGCTTTGGATGACGT
RANKL	F: AGGAGGTGAAGTTAATGCCAGAAT R: AGTTTCCCATCACTGAACGT CATA

### Western blot assay

We performed Western blot analysis as previously described (3). Tibial growth plate specimens were centrifuged, and the supernatants were separated by SDS-polyacrylamide gel electrophoresis and blotted onto a nitrocellulose membrane (Millipore). The membranes were incubated with rabbit-source antibodies to FGF2 (Abclonal Technology, #A0235, 1:1,000), FGFR1 (Abclonal Technology, #A2073, 1:1,000), VEGFA (Abcam, #ab46154, 1:1,000) and VEGFR1 (Abcam, #ab32152, 1:1,000), and then re-probed with appropriate horseradish peroxidase-conjugated secondary antibodies (Servicebio technology, #GB23303, 1:3,000). The membranes were then visualized by enhanced chemiluminescence (ECL Kit, Servicebio technology, #G2014) and exposed to X-ray films.

### Enzyme-linked immunosorbent assay (ELISA)

All blood samples were centrifuged at 3,500 rpm for 10 min, and the supernatants were stored at −80 °C until use. VEGFA, VEGFR1, VEGFR2, Ang1, Tie2, PDGF-BB, PDGFR-β, OPG and RANKL levels in chicken sera were assayed using a specific ELISA kit according to the manufacturer’s recommendations. The Chicken-specific VEGFA ELISA kit (Biofine, #E75113), chicken-specific VEGFR1 ELISA kit (Biofine, #E75019) and chicken-specific VEGFR2 ELISA kit (Biofine, #E75148) were purchased from Equation Jiahong Technology (Beijing, China). The Chicken-specific Ang1 ELISA kit (Elisa Lab, #JYM0119Ch), chicken-specific Tie2 ELISA kit (Elisa Lab, #JYM0118Ch), chicken-specific PDGF-BB ELISA kit (Elisa Lab, #JYM0127Ch), chicken-specific PDGFR-β ELISA kit (Elisa Lab, #JYM0121Ch), chicken-specific OPG ELISA kit (Elisa Lab, #JYM0132Ch), and chicken-specific RANKL ELISA kit (Elisa Lab, #JYM0131Ch) were purchased from ColorfulGene Biological Technology (Wuhan, China). The optical density values of each well were determined within 15 min at a wavelength of 450 nm using a Thermo Scientific Microplate Reader (Multiskan® MK3).

### Statistical analysis

Data are presented as the means ± s.d. or s.e.m. Correlation analyses were performed using Spearman tests, comparisons between two groups were analyzed using unpaired, two-tailed Student’s *t*-tests, and comparisons of multiple groups were analyzed using one-way analysis of variance (ANOVA) followed by Fisher’s least significant difference (LSD) testing with SPSS (Version 17.0. Chicago, IL) or Prism (#Version 7.00, GraphPad Software, Inc.) statistical analysis software. All experiments were repeated at least three times to increase the reproducibility of the findings, and representative experiments are shown. *P* < 0.05 was considered to be statistically significant.

## Electronic supplementary material

Supplementary Information
