# Supplementary material for: Role and regulation of growth plate vascularization during coupling with osteogenesis in tibial dyschondroplasia of chickens
Source: Sci Rep. 2018 Feb 27;8:3680. doi: 10.1038/s41598-018-22109-y (PMC5829164; doi:10.1038/s41598-018-22109-y)

Role and regulation of growth plate vascularization during coupling with osteogenesis in tibial dyschondroplasia of chickens

Shu-cheng Huang^1^, Li-hong Zhang^1^, Jia-lu Zhang^1^, Mujeeb Ur Rehman^1^, Xiao-le Tong^1^, Gang Qiu^1,2^, Xiong Jiang^1,3^, Mujahid Iqbal^1^, Muhammad Shahzad^4^,Yaoqin Shen^1*^, Jiakui Li^1,2*^

**Supplementary Figure 1. The changes in blood parameters during the growth of broiler chickens** (**g-j**) Quantitative analysis of growth rate and increment speed of blood parameters (including RBC, HCT, Hb, MCV, MCH, and MCHC) in the normal group from 7-, 10 and 14-day-old broiler chickens. N=8 chickens in each group from four independent experiments; Data represent means ± s.d. ^*^*p*<0.05, ^**^*p*<0.01, ^***^*p*<0.001, one-way analysis of variance (ANOVA) and Least-significant difference (LSD) Duncan test. NS, not significant; RBC, red blood cell counts; Hb, hemoglobin; Hct, hematocrit; MCV, mean corpuscular volume; MCH, mean corpuscular hemoglobin; MCHC, mean corpuscular hemoglobin concentration.





**Supplementary Figure 2. The changes in vasculogenesis-related serum protein levels. (a, b)** VEGFR1/VEGFA ratios and VEGFR2/VEGFA ratios were determined by ELISA in serum. (n=9 in each group from three independent experiments, 3 technical replicates per experiment). Data represent means ± s.d. ^**^*p*<0.01, ^***^*p*<0.001, two-tailed unpaired *t*-test. NS, not significant.


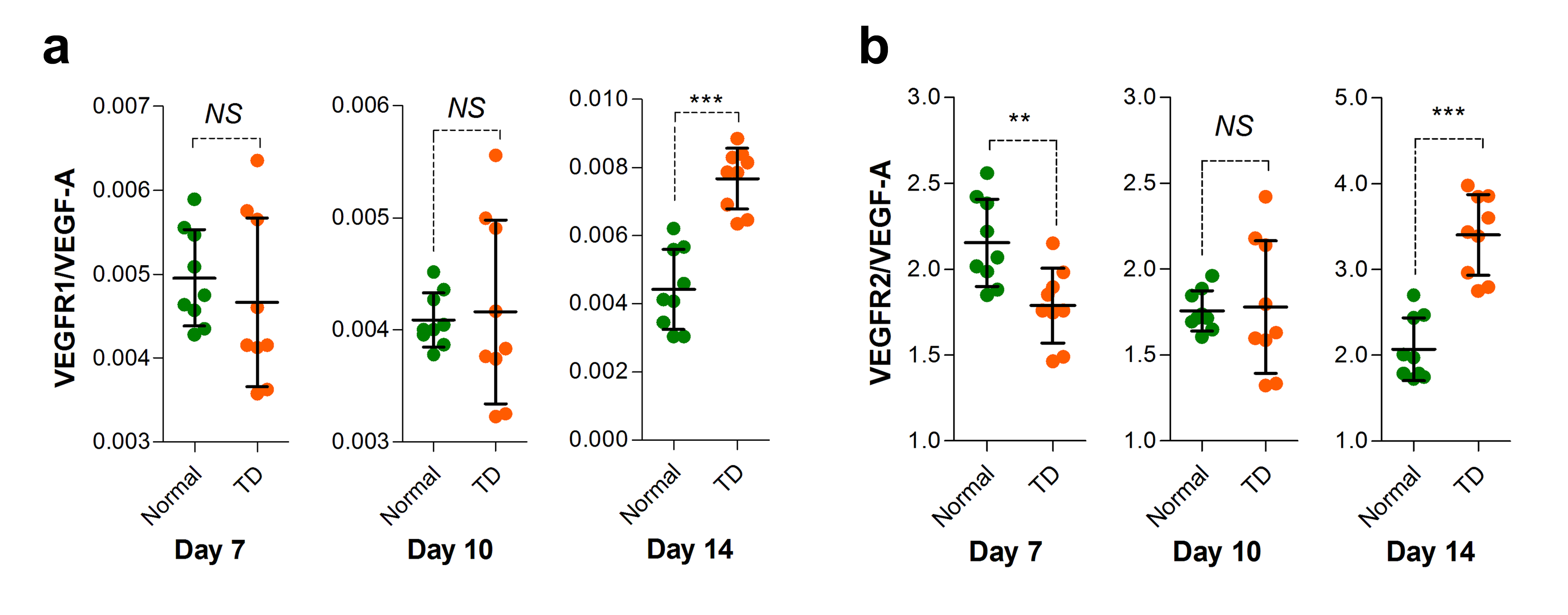


**Supplementary Figure 3. The changes in blood parameters in TD chickens.** (**a-c**) Blood MCV, MCH, and MCHC were assessed in normal and TD chickens. Data represent means ± s.e.m. (n=4 or 6 chickens from three independent experiments). ^*^*p*<0.05, ^**^*p*<0.01, two-tailed unpaired *t*-test. NS, not significant; MCV, mean corpuscular volume; MCH, mean corpuscular hemoglobin; MCHC, mean corpuscular hemoglobin concentration.


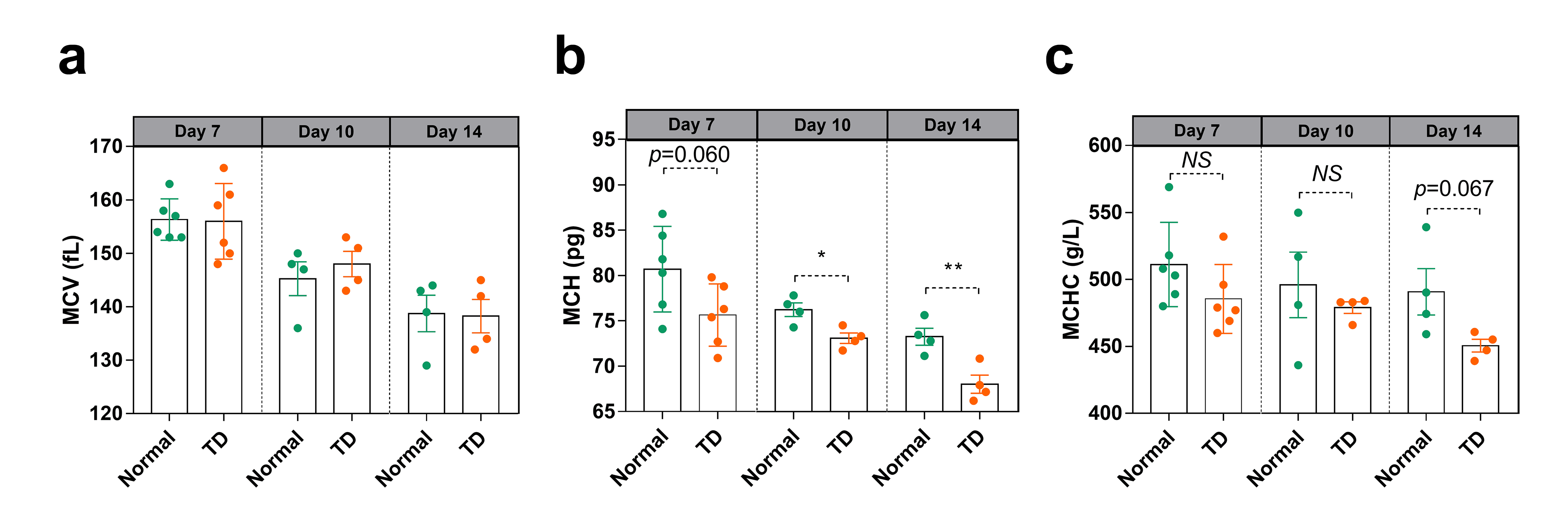


**Supplementary Figure 4. Evaluation of the correlation between tibia parameters and gene expression. (a-d)** Correlation analysis between TW and serum OPG level (**a**), between TL and serum OPG level (**b**), between TW and serum RANKL level (**c**), and between TL and serum RANKL level (**d**) in normal and TD chickens was performed by Spearman tests. n=8 chickens for tibia weight and tibia length; n=9 in each group from three independent experiments, 3 technical replicates per experiment for serum OPG and RANKL levels.


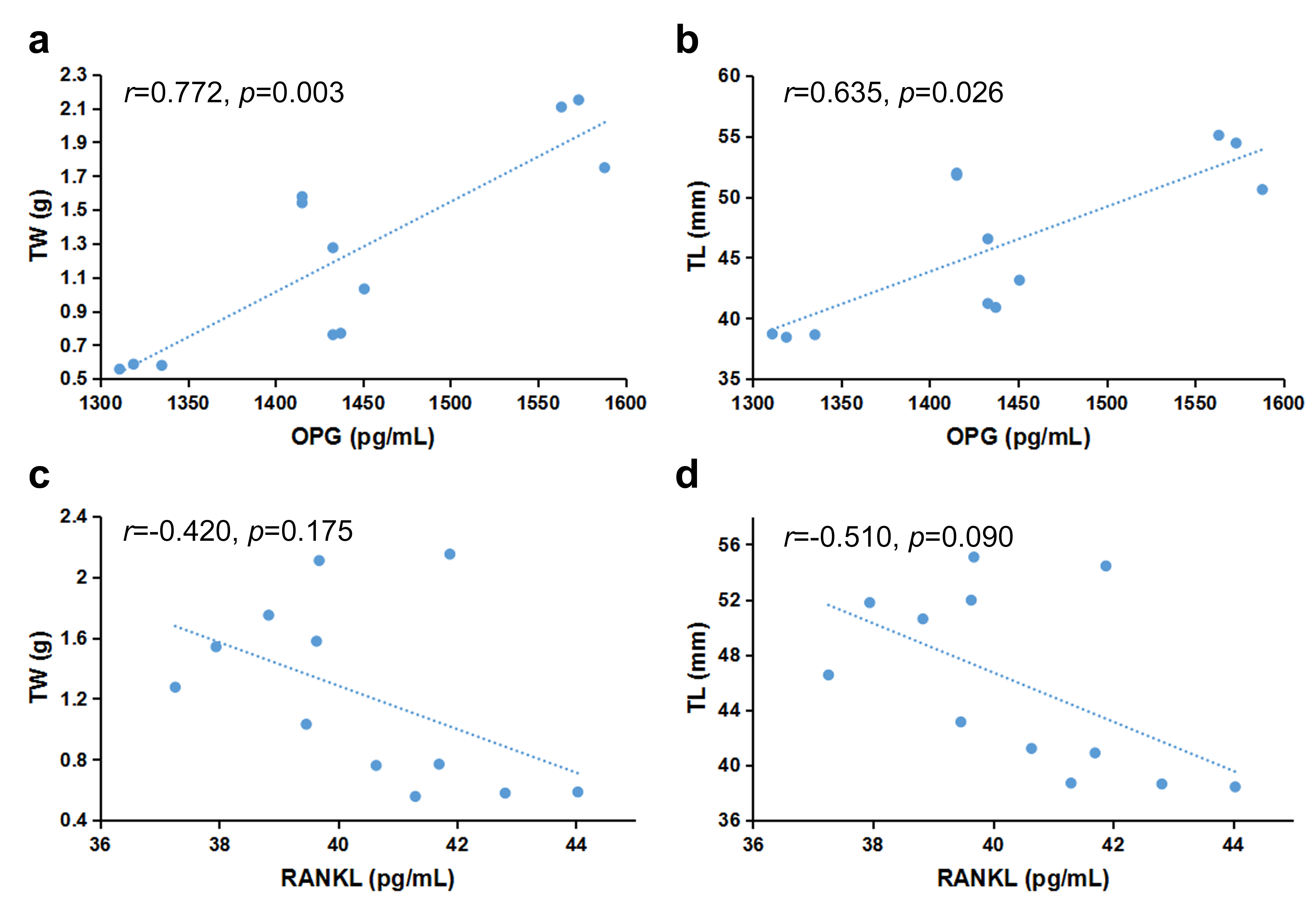

Supplement: Supplementary file 1 — Supplementary Information [file 41598_2018_22109_MOESM1_ESM.doc]
